# COVID-19 lockdown and the tobacco product ban in South Africa

**DOI:** 10.18332/tid/120938

**Published:** 2020-05-06

**Authors:** Catherine O. Egbe, Senamile P. Ngobese

**Affiliations:** 1 Alcohol, Tobacco and Other Drug Research Unit, South African Medical Research Council, Pretoria, South Africa; 2 Department of Public Health, Sefako Makgatho Health Sciences University, Pretoria, South Africa

**Keywords:** South Africa, COVID-19, tobacco product use, lockdown, sales ban on tobacco products

**Dear Editor,**

South Africa (SA) imposed a 21-day lockdown from 26 March to 16 April 2020, through the disaster management Act (57/2002) and amended regulations [Section 27(2)]^1^, to contain the spread of the coronavirus in the country. During the lockdown only essential goods have been allowed on sale^2^. Tobacco and nicotine products were designated as non-essential goods and their sales prohibited.

About 22% of South Africans aged ≥15 years use various tobacco products^3^. A systematic review of five Chinese studies published in Tobacco Induced Diseases, found that the odds of a COVID-19 case becoming more severe and leading to death are higher among people with a history of smoking^4^. SA has one of the highest prevalence of tobacco product use in Africa^5^ and presently has the highest number of COVID-19 cases in the continent, putting the country at risk of being hard hit by the COVID-19 pandemic if nothing is done to curb the spread of the virus and protect vulnerable citizens. As of 8 April 2020, SA had conducted 63776 COVID-19 tests, had 1845 confirmed cases and 18 resulting deaths^6^.

The COVID-19 pandemic could particularly be problematic for SA given the high prevalence of diabetes, tuberculosis and HIV in the country, compounded by substance use problem even among vulnerable populations^7^. The co-use of substances, especially by persons in vulnerable groups, could increase the risk of developing complications from COVID-19 if infected.

Cigarette smoking affects both smokers and those exposed to secondhand smoke (SHS)^8^. During the lockdown ban, family members and neighbors in apartment complexes who share the same space with tobacco users will also be protected from exposure to SHS since people are not expected to go outside their building to smoke during the lockdown. Experts have predicted that the South African economy is in for a bumpy ride and may shrink as a result of the COVID-19 pandemic^9^ and the associated measures that have been taken to protect citizens during this time.

The government has received criticism from some pro-tobacco advocates and academics for the sales ban on tobacco products and alcohol during the lockdown^10^, but this move is heroic and should be viewed as taking a double shot at protecting citizens from COVID-19, given the vulnerability of many South Africans to the disease. There are also concerns about the implications on mental health when people are forced to give up their addictions during the lockdown^10^. It is understandable that tobacco and nicotine product users will be dealing with the mental stress of restricted movements in addition to giving up their addictions, but of more importance is that they become aware that the lockdown ban provides a good opportunity to quit tobacco use. In addition, resources to help tobacco users quit, or assist them to cope with withdrawals, should be made available and accessible. The National Council Against Smoking’s quitline (011 720 3145), online materials (https://www.againstsmoking.co.za/) and the Cancer Association of South Africa’s online quit tobacco program (https://www.ekickbutt.org.za/) are some good resources accessible to tobacco users to help them quit.
